# Subcellular Localization Dictates Therapeutic Function: Spatially Targeted Delivery of Amuc_1100 by Engineered *Lacticaseibacillus paracasei* L9 Enhances Intestinal Barrier in Colitis

**DOI:** 10.3390/nu18010123

**Published:** 2025-12-30

**Authors:** Xinrui Dong, Li Lin, Weina Miao, Zhengyuan Zhai, Yanling Hao, Ming Zhang, Ran Wang, Shaoyang Ge, Hao Zhang, Lianzhong Ai, Liang Zhao

**Affiliations:** 1 College of Food Science and Nutritional Engineering, China Agricultural University, Beijing 100083, China; s20213061014@cau.edu.cn (X.D.); linli@cau.edu.cn (L.L.); s20233061205@cau.edu.cn (W.M.); zhaizy@cau.edu.cn (Z.Z.); zhanghaocau@cau.edu.cn (H.Z.); 2 Key Laboratory of Functional Dairy, Department of Nutrition and Health, China Agricultural University, Beijing 100193, China; haoyl@cau.edu.cn (Y.H.); wangran@cau.edu.cn (R.W.); 3 School of Food and Health, Beijing Technology and Business University, Beijing 100048, China; zhangming@th.btbu.edu.cn; 4 Research Center for Probiotics, China Agricultural University, Beijing 101299, China; geshaoyang@foxmail.com; 5 Shanghai Engineering Research Center of Food Microbiology, School of Health Science and Engineering, University of Shanghai for Science and Technology, Shanghai 200093, China; ailianzhong@163.com; 6 School of Agriculture and Biology, Shanghai Jiao Tong University, Shanghai 200240, China

**Keywords:** Amuc_1100 protein, *Lacticaseibacillus paracasei*, inflammatory bowel disease, engineered probiotics, *Akkermansia muciniphila*, intestinal barrier

## Abstract

**Background/Objectives**: Impaired intestinal barrier function is a hallmark of inflammatory bowel disease (IBD). *Akkermansia muciniphila* and its outer membrane protein Amuc_1100 can enhance this barrier, but the clinical application of Amuc_1100 is limited by the fastidious growth of its native host. This study aimed to overcome this by utilizing the robust probiotic *Lacticaseibacillus paracasei* L9 for targeted Amuc_1100 delivery. **Methods**: We engineered *Lc. paracasei* L9 to express Amuc_1100 via intracellular (pA-L9), secretory (pUA-L9), and surface-display (pUPA-L9) strategies. Their efficacy was assessed in Lipopolysaccharide (LPS)-induced macrophages and a dextran sulfate sodium (DSS)-induced colitis mouse model, evaluating inflammation, barrier integrity, and mucosal repair. **Results**: The secretory (pUA-L9) and surface-display (pUPA-L9) strains most effectively suppressed pro-inflammatory cytokines (*IL-6*, *IL-1β*, and *TNF-α*) in macrophages. In mice, both strains alleviated colitis and outperformed native *A. muciniphila* in improving disease activity. Crucially, they exhibited distinct, specialized functions: pUA-L9 acted as a systemic immunomodulator, reducing pro-inflammatory cytokines (*IL-6*, *IL-1β*, and *TNF-α*), elevating anti-inflammatory mediators (*IL-4* and *IL-10*), and promoting goblet cell differentiation; notably, the inhibitory effect of pUA-L9 on *IL-6* expression was approximately 2-fold greater than that of pUPA-L9. In contrast, pUPA-L9 excelled in local barrier repair, uniquely restoring mucus layer integrity (*Muc1*, *Muc2*, and *Tff3*) and reinforcing tight junctions (*ZO-1*, *Occludin*, *Claudin1*, *Claudin3*, and *Claudin4*). In particular, pUPA-L9 increased *Muc2* expression by approximately 3.6-fold compared with pUA-L9. **Conclusions**: We demonstrate that the subcellular localization of Amuc_1100 within an engineered probiotic dictates its therapeutic mode of action. The complementary effects of secretory and surface-displayed Amuc_1100 offer a novel, spatially targeted strategy for precision microbiome therapy in IBD.

## 1. Introduction

Inflammatory bowel disease (IBD) encompasses a group of chronic, idiopathic inflammatory disorders of the gut, primarily including ulcerative colitis (UC) and Crohn’s disease (CD) [1,2]. According to Global Burden of Disease (GBD) data, by 2021, the prevalence of IBD reached 3.83 million cases, with incidence increasing in 167 countries globally. This presents significant challenges for clinical practice, particularly as chronic inflammatory stimulation is closely linked to the development of colon cancer [3]. A defining pathological feature of IBD is intestinal barrier dysfunction, characterized by disruption of the mucus layer and impairment of epithelial tight junctions, which facilitates microbial translocation and perpetuates mucosal inflammation [4,5,6,7,8,9,10]. Restoration of barrier integrity has therefore emerged as a central therapeutic strategy in IBD management [11,12]. The core gut probiotics—such as *Bifidobacterium*, *Lactobacillus*, *Clostridium butyricum* [13,14], *A. muciniphila*, and short-chain fatty acid (SCFA)-producing bacteria, including *Faecalibacterium prausnitzii* and *Roseburia* spp. [15,16]—significantly contribute to the maintenance of intestinal barrier integrity. Among these, *A. muciniphila*, a key mucin-degrading bacterium, plays a significant role in maintaining intestinal barrier function and regulating host metabolism [17].

*A. muciniphila* degrades intestinal mucin via the action of glycosidases, proteases, and sialidases [18,19], metabolizes SCFAs, and provides energy to intestinal goblet cells. This metabolic activity contributes to an increase in the thickness of the intestinal mucus layer, thereby enhancing the overall integrity of the intestinal barrier [20,21]. A recent study reported that *A. muciniphila* subjected to pasteurization exhibits a greater ability to improve metabolic diseases compared to its active bacterial form [22]. This suggests that the functional components of *A. muciniphila* may be crucial to its probiotic effects. Amuc_1100, a 32 kDa outer membrane protein encoded by a type IV pilus gene cluster, is the most abundant and well-characterized protein of *A. muciniphila* [23]. As a postbiotic, Amuc_1100 exerts multiple health-regulating effects, including alleviation of intestinal inflammation and enhancement of barrier function through modulation of the CREBH–IGF signaling pathway [24]. In addition, Amuc_1100 displays dual immunomodulatory activities by regulating inflammatory cytokine production via Toll-like receptors 2 and 4 (TLR2/TLR4) [25] and reshaping the intestinal immune microenvironment through upregulation of PD-1 expression, suppression of CD8^+^ cytotoxic T lymphocyte activation, and reduction in pro-inflammatory macrophage populations [26]. Owing to these properties, Amuc_1100 has emerged as a promising postbiotic candidate with pharmaceutical and nutraceutical potential. However, its practical application is limited by the strict anaerobic growth requirements of *A. muciniphila* and its dependence on mucin as the sole nutrient source, leading to complex purification procedures and high production costs [27]. As a result, heterologous expression has become the primary strategy for Amuc_1100 production, with *Escherichia coli* being the most commonly used expression system in experimental studies [28,29]. However, the high purification costs, potential chemical residues, and biosafety risks associated with this production method make it unsuitable for large-scale industrial applications in food and drug production.

Moreover, the location of protein expression in engineered strains significantly impacts its efficacy. Intracellular expression of functional proteins can mitigate environmental interference, enhance yield and stability, and is frequently utilized for industrial enzyme production and protein storage [30,31]. In contrast, secreted expression facilitates correct protein folding by releasing proteins into the extracellular space, thereby promoting precise functionality; however, this approach is limited by poor resistance to external environmental conditions. Surface display systems, which promote the adhesion properties and immune antigenicity of recombinant strains, are primarily used in vaccine development [32,33], but this method may induce metabolic stress and perturb the membrane structure of the vector. Therefore, a strategic selection of the expression system is essential, tailored to specific needs and applications.

In recent years, probiotics have been extensively studied as carriers for the delivery of functional proteins. As a group of microorganisms that have coexisted with humans in food systems and the gastrointestinal tract for millennia, the vast majority of lactic acid bacteria are classified as Generally Recognized As Safe (GRAS) by regulatory authorities [34]. Additionally, their typical utilization in live bacterial form enables targeted delivery to the intestinal tract, allowing them to integrate and exert synergistic effects with exogenous functionalities while retaining their inherent probiotic properties [35,36]. *Lc. paracasei* L9 is a lactic acid bacterium isolated from the intestines of elderly individuals, known for its various physiological regulatory effects. Notably, it demonstrates resilience to acid and bile salt stress to a certain extent [37,38], which provides a distinct advantage for its application as a probiotic. Previous studies have indicated that *Lc. paracasei* L9 can enhance the abundance of butyrate-producing bacteria, thereby inhibiting the IL-6/STAT3 signaling pathway and alleviating the development of DSS-induced colitis [39]. Additionally, *Lc. paracasei* L9 has been shown to bolster innate immunity by activating TLR2 in the mucosal layer, while also inducing macrophages to produce *IL-12*, which promotes Th1 polarization and thereby enhances acquired immunity [40]. Therefore, *Lc. paracasei* L9 serves as a superior vector for Amuc_1100 expression, enabling targeted delivery of the protein to the host colon and facilitating synergistic functional enhancement.

While the therapeutic potential of Amuc_1100 is evident, its optimal delivery platform remains unexplored. Heterologous expression in *E. coli* faces challenges in biosafety and purification cost, limiting its application in functional foods or live biotherapeutics. Furthermore, the functional outcome of a protein therapeutic is often dictated by its spatial presentation and accessibility in the gut environment. To date, however, few studies have systematically compared multiple protein expression and localization strategies within the same probiotic chassis to determine how subcellular localization shapes distinct systemic versus local therapeutic outcomes *in vivo*. We hypothesized that the subcellular localization of Amuc_1100 within a probiotic vector would critically influence its therapeutic efficacy and functional specificity. To test this, we engineered *Lacticaseibacillus paracasei* L9—a strain with documented gut resilience and beneficial properties—to deliver Amuc_1100 via three distinct strategies: intracellular retention (pA-L9), extracellular secretion (pUA-L9), and surface display (pUPA-L9), and systematically evaluated their effects on inflammation and intestinal barrier integrity using LPS-stimulated macrophages and a DSS-induced colitis mouse model. This comparative approach was designed to provide a scalable production strategy and to explore whether distinct subcellular localization of Amuc_1100 could dissociate systemic immunomodulatory effects from localized intestinal barrier–repair functions, with the aim of informing the development of precision microbiome-based therapies for IBD.

## 2. Materials and Methods

### 2.1. Bacterial Strains, Plasmids, and Culture Conditions

Bacterial strains and plasmids used in this study are listed in Table S1. *Lc. paracasei* L9 (CGMCC NO. 9800) was cultured in MRS (Land Bridge, Beijing, China) broth at 37 °C for 12 h. *Lactococcus lactis* NZ9000 (*L. lactis* NZ9000) was statically cultured in M17 (Oxoid, Unipath, Basingstoke, UK) broth, supplementing 0.5% (*w*/*v*) glucose (GM17) at 30 °C for 8 h. *A. muciniphila* ATCC BAA-835 was grown in BHI (Land Bridge, Beijing, China) broth, supplementing 0.05% (*w*/*v*) cysteine in an anaerobic chamber at 37 °C for 48 h. When required, the medium was supplemented with 10 μg/mL erythromycin (Solarbio, Beijing, China) for *Lc. paracasei* L9 and *L. lactis* NZ9000.

### 2.2. Construction and Expression of Recombinant Bacteria

The gene sequence of the Amuc_1100 protein was retrieved from the National Center for Biotechnology Information. A codon-optimized version of the Amuc_1100 gene (approximately 880 bp), excluding the signal peptide, was synthesized de novo (Beijing Tsingke Biotechnology Co., Ltd., Beijing, China). Three sets of primers (Am-F-411 and Am-R-411, Am-F-usp45 and Am-R-411, Am-F-pgsA’ and Am-R-411) were used to amplify three segments of the Amuc_1100 protein gene sequence with different connecting gene homologous arms (Table S2). The signal peptide gene sequence of *Lactococcus lactis* MG1363 usp45 (approximately 80 bp) and the truncated pgsA’ gene sequence of *Bacillus subtilis* subsp. 168 (approximately 420 bp) were also synthesized. Three sets of primers (usp45-F-411 and usp45-R-Am, usp45-F-411 and usp45-R-pgsA’, pgsA’-F-usp45 and pgsA’-R-Am) were used to clone the usp45 and pgsA’ gene sequences with different connecting gene homologous arms (Table S2). The amplified gene sequences were cloned into the *NcoI* and *HindIII* (both from NEB, Beijing, China) double-digested pSIP411 plasmid using the ClonExpress Ultra One Step Cloning Kit (Vazyme, Nanjing, China) to construct three expression vectors for intracellular expression, secretory expression, and surface display of the Amuc_1100 protein.

The recombinant plasmids were then transformed into *L. lactis* NZ9000 by electroporation using Bio-Rad Gene Pulser Xcell (Bio-Rad, Richmond, CA, USA) as previously described [41]. The correct recombinant plasmid was identified by DNA sequencing (Sangon Biotech, Shanghai, China) [42]. The correct recombinant plasmids were then transformed into *Lc. paracasei* L9 by electroporation. All recombinant bacteria were selected on MRS agar plates containing 10 µg/mL erythromycin. Expression of the Amuc_1100 protein was verified via Western blotting analysis.

### 2.3. Immune Colloidal Gold Transmission Electron Microscopy

After 48 h of cultivation, the bacterial culture medium was collected and centrifuged at 6000 rpm for 10 min at 4 °C using a Sorvall LYNX 4000 high-speed centrifuge (Thermo Fisher Scientific, Waltham, MA, USA) to harvest the bacterial precipitate. Subsequently, the cells were pre-fixed at 4 °C for 15 min using an immunoelectron microscopy fixative. After fixation, the cells were blocked with phosphate-buffered saline (PBS, Solarbio, Beijing, China) supplemented with 5% bovine serum albumin (BSA, Beyotime, Shanghai, China). The cells were incubated with a mouse anti-6×His IgG2b primary antibody (diluted 1:100; BioLegend, San Diego, CA, USA) and a mouse-specific secondary antibody conjugated to a colloidal gold (BSZH Scientific, Beijing, China). The bacteria were centrifuged again at 3000 rpm for 15 min to collect the pellet. Finally, samples underwent standard embedding and staining procedures before Transmission Electron Microscopy observation [43].

### 2.4. Effect of Recombinant Strain on Pro-Inflammatory Response in Macrophages

RAW264.7 cells (purchased from the Cell Bank, Chinese Academy of Sciences, Shanghai, China) were maintained in Dulbecco’s Modified Eagle Medium (DMEM, Gibco, Thermo Fisher Scientific, Waltham, MA, USA) supplemented with 10% (*v*/*v*) Fetal Bovine Serum (FBS, Biological Industries, Kibbutz Beit Haemek, Israel), 100 U/mL penicillin, and 100 μg/mL streptomycin (Beyotime, Shanghai, China) at 37 °C in a humidified incubator (3111, Thermo Fisher Scientific, Waltham, MA, USA) with 5% CO_2_. For experiments, cells were seeded at a density of 1 × 10^5^ cells per well in 12-well plates and starved in serum-free DMEM for 12 h prior to treatment. The experimental groups were as follows: cells treated with DMEM complete medium without the addition of LPS and bacteria (Control), model cells treated with LPS but without bacteria (LPS), experimental cells treated with LPS and *Lc. paracasei* L9 with empty vector (L9), experimental cells treated with LPS and *A. muciniphila* (AKK), experimental cells treated with LPS and *Lc. paracasei* L9 expressing Amuc_1100 protein within cells (pA-L9), experimental cells treated with LPS and *Lc. paracasei* L9 secreting Amuc_1100 protein (pUA-L9), experimental cells treated with LPS and *Lc. paracasei* L9 displaying Amuc_1100 protein on surface(pUPA-L9). Subsequently, DMEM complete medium containing LPS (final concentration: 500 ng/mL) was added to each well (except for the Control group), and cells were incubated for 4 h under standard culture conditions (37 °C, 5% CO_2_). Recombinant bacteria to be added to each experimental group were cultured for 12 h. Subsequently, a 1 mL aliquot (1 × 10^9^ CFU/mL) of live recombinant bacteria from each group was collected, washed three times with Hank’s Balanced Salt Solution (HBSS, Solarbio, Beijing, China), and resuspended in HBSS. The bacteria were then introduced to LPS-induced RAW264.7 cells (4 h induction) and incubated at 37 °C for 30 min. Upon completion of the experiment, cell samples from each group were collected for subsequent analysis.

### 2.5. Animal Experiment Design

All experimental procedures were approved by the Institutional Animal Care and Use Committee (IACUC) of Sinoresearch (Beijing) Biotechnology Co., Ltd. (Approval No. ZYZC2024070002S) and were conducted in strict accordance with the internationally recognized Guidelines for the Care and Use of Laboratory Animals (National Research Council, 8th edition, 2011). Male 6- to 8-week-old male mice (Purchased from Beijing Vital River Laboratories Technology Co., Ltd., Beijing, China) were housed in a specific pathogen-free (SPF) barrier facility under controlled conditions (12/12 h light/dark cycle, 21 ± 1 °C ambient temperature, 50 ± 5% relative humidity) with free access to water and food. After a 7-day acclimatization period, mice were weighed and randomly allocated to experimental groups using stratified block randomization to balance baseline body weight across groups. Specifically, animals were ranked by body weight and assigned in blocks of six (one mouse per group per block) using a computer-generated random sequence. After randomization, mice were assigned to six experimental groups (*n* = 6 biologically independent animals per group) as follows: healthy mice consumed regular water (Control), model mice consumed 2.5% DSS (DSS), experimental mice consumed 2.5% DSS and were administered *Lc. paracasei* L9 with empty vector (L9), experimental mice consumed 2.5% DSS and were administered *A. muciniphila* (AKK), experimental mice consumed 2.5% DSS and administered *Lc. paracasei* L9 secreting Amuc_1100 protein (pUA-L9), experimental mice consumed 2.5% DSS and were administered *Lc. paracasei* L9 displaying Amuc_1100 protein on surface (pUPA-L9). From days 0 to 21, mice of the Control and DSS groups were administered with PBS, while the L9, AKK, pUA-L9 and pUPA-L9 groups were pretreated with 200 µL 5 × 10^9^ CFU per 200 μL live *Lc. paracasei* L9 with empty vector, *A. muciniphila*, *Lc. paracasei* L9 secreting Amuc_1100 protein and *Lc. paracasei* L9 displaying Amuc_1100 protein on surface suspended in PBS, respectively. From days 14 to 21, the mice (except for the Control group) were supplied with drinking water containing 2.5% DSS (freshly prepared daily) to induce IBD (Figure S2). During the experiment, all mice were observed daily for body weight, Disease Activity Index (DAI), and general condition. The body weight (BW) loss of each mouse was expressed as a percentage relative to its baseline weight before DSS administration. Specifically, the DAI was assessed each day based on weight loss, stool consistency, and fecal occult blood, following previously described methods [44] (Table S3). DAI scoring was performed in a blinded manner by two independent investigators who were unaware of group allocation, and any discrepancies were resolved by consensus. Twelve hours prior to the end of the experiment, mice were fasted (food withheld). Following euthanasia via carbon dioxide asphyxiation, spleen and colon tissues were collected. Colonic tissues were fixed in 4% paraformaldehyde and Carnoy’s solution (Servicebio, Wuhan, China) for histological evaluation. Based on previous studies [45], evaluations were conducted according to the severity of inflammation, the extent of inflammation, and the degree of crypt damage. The detailed scoring criteria for colonic histopathology are summarized in Table S4. Histological scoring was performed in a blinded manner by two independent investigators, and discrepancies were resolved by consensus. PAS-positive cells were counted manually in well-oriented colonic sections by two independent investigators blinded to group allocation. For each animal, at least five randomly selected, non-overlapping fields were analyzed under ×200 magnification, and goblet cell numbers were normalized to the length of the epithelium (cells per mm). The average value from each animal was used for statistical analysis. The remaining tissue samples were immediately stored at −80 °C until analysis.

### 2.6. Quantitative Real-Time PCR Analysis

Total RNA was extracted from RAW264.7 cells and frozen mouse colon tissue using TRIzol reagent (Tiangen, Beijing, China). The integrity of the RNA samples was assessed using an Infinite 200 PRO microplate reader (Tecan, Männedorf, Switzerland). The extracted RNA was reverse-transcribed into cDNA using the All-In-One Reverse Transcription Kit (abm, Vancouver, BC, Canada). Quantitative real-time PCR was performed on a Roche LightCycler instrument (Roche, Basel, Switzerland) using the SYBR Green fluorescent dye method to quantify gene expression. The results were calculated using the 2^−ΔΔCt^ method, with GAPDH serving as the internal reference gene. The primers were synthesized by Sangon Bioengineering Co., Ltd., Shanghai, China, and the details are provided in Tables S5 and S6.

### 2.7. Western Blot

Four recombinant strains, L9, pA-L9, pUA-L9 and pUPA-L9 (employed for detecting the expression of recombinant proteins), as well as mouse colon tissue samples (used to examine protein expression in colon tissues), which had reached the stationary phase of growth, were homogenized in a cell and tissue lysis buffer supplemented with protease and phosphatase inhibitors (Servicebio, Wuhan, China). The total protein concentration was determined using a BCA kit (Soleibao, Beijing, China). Subsequently, proteins were separated by SDS-PAGE gel electrophoresis and transferred onto a polyvinylidene fluoride (PVDF) membrane (0.45 μm, IPVH00010, Millipore, Beijing, China). The membrane was blocked with 5% bovine serum albumin (BSA, Beyotime, Shanghai, China) for 2 h. Then, it was incubated overnight at 4 °C with an anti-His-Tag mouse monoclonal antibody (1:5000 dilution; BioLegend, CA, USA), as well as NF-κB and pNF-κB rabbit monoclonal antibodies (1:5000 dilution; CST, Danvers, MA, USA). Afterward, the membrane was washed three times with TBST and incubated at room temperature for 1 h with HRP-conjugated goat anti-mouse IgG antibody (1:5000 dilution; Abcam, Waltham, MA, USA) and HRP-conjugated goat anti-rabbit IgG antibody (1:5000 dilution; Zsbio, Beijing, China). Finally, detection was carried out using enhanced chemiluminescence (ECL) reagent (Epizyme, Shanghai, China). Protein bands were visualized using an appropriate detection system (Amersham Imager 600, GE Healthcare, Chicago, IL, USA). The density of the protein bands was analyzed with ImageJ analysis software (version 1.53, National Institutes of Health, Bethesda, MD, USA), and protein abundance was normalized against β-actin.

### 2.8. Statistical Analysis

All data are presented as mean ± standard deviation (SD). Data were tested for normality using the Shapiro–Wilk test and for homogeneity of variances prior to statistical analysis. When these assumptions were satisfied, differences among multiple groups were analyzed using one-way analysis of variance (ANOVA). Post hoc comparisons were performed using Duncan’s multiple range test to identify differences between groups. Statistical analyses were conducted using SPSS 26.0 (IBM Corp., Armonk, NY, USA). A *p* value < 0.05 was considered statistically significant.

## 3. Results

### 3.1. Expression and Detection of Amuc_1100 in Lc. paracasei L9 Recombinant Strains

**Figure 1:**
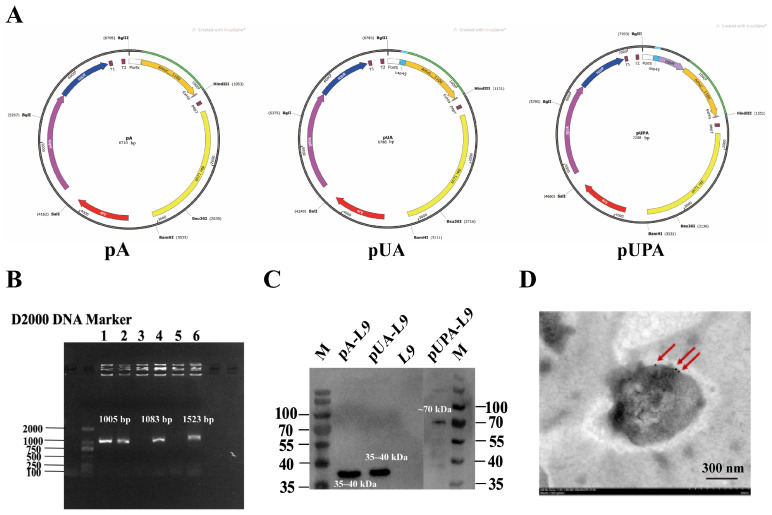
Expression and detection of Amuc_1100 in *Lc. paracasei* L9. (**A**) Schematic diagram of recombinant vectors. (**B**) Gel electrophoresis of PCR products from recombinant plasmids. A 1% TAE agarose gel was used, with 3 μL of sample loaded per well. Lanes 1 and 2 contained recombinant plasmids for intracellular (pA-L9), lanes 3 and 4 for secretory (pUA-L9), and lanes 5 and 6 for surface-display (pUPA-L9). (**C**) Western blot assay results for recombinant protein. Line 1 and 6 contained protein marker (Epizyme, Shanghai, China), line 2 contained recombinant plasmids for intracellular (pA-L9), line 3 for secretory (pUA-L9), line 4 for *Lc. paracasei* L9 with empty vector, and line 5 for surface-display (pUPA-L9). (**D**) TEM image of surface-displayed Amuc_1100 following immunogold labeling. The arrow indicates Amuc_1100 protein conjugated with antibody-labeled colloidal gold particle.

To evaluate the expression of Amuc_1100 in *Lc. paracasei* L9, three recombinant vectors—intracellular (pA-L9), secretory (pUA-L9), and surface-display (pUPA-L9)—were constructed and transformed into the host strain. Positive recombinant clones selected on erythromycin-containing plates were verified by plasmid sequencing, which confirmed correct insertions. All recombinant plasmids were verified by full-length Sanger sequencing of the inserted regions to confirm correct assembly, in-frame fusion, and sequence fidelity, with no mutations detected. Electrophoretic analysis of extracted plasmids revealed successful vector integration in lanes 1, 2, 4, and 6 (Figure 1B), corresponding to pA-L9 (intracellular), pUA-L9 (secretory), and pUPA-L9 (surface-display) constructs.

Western blot analysis demonstrated successful Amuc_1100 expression across all three systems (Figure 1C). Specifically, the protein was detected in cell lysates of pA-L9 (intracellular expression), culture supernatants of pUA-L9 (secretory expression), and cell wall fractions of pUPA-L9 (surface-display).

To confirm surface localization of Amuc_1100 in pUPA-L9 (surface-display) recombinants, immunogold labeling coupled with transmission electron microscopy (TEM) was performed. Distinct gold particle labeling on the bacterial surface (Figure 1D) confirmed that Amuc_1100 was successfully anchored to the bacterial surface via the pUPA-L9 system.

Taken together, these findings highlight that *Lc. paracasei* L9 efficiently expressed and localized Amuc_1100 via intracellular, secretory, and surface-display mechanisms.

### 3.2. Anti-Inflammatory Effects of Lc. paracasei L9 Recombinant Strains on Macrophages

**Figure 2:**
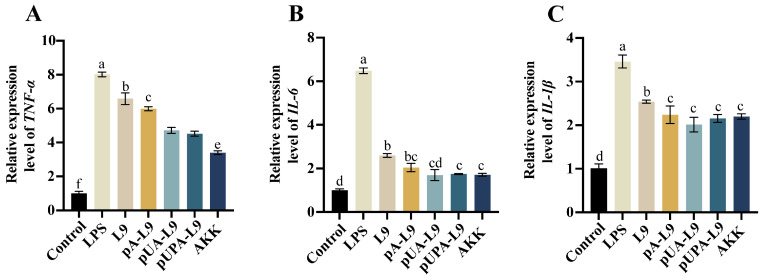
Effect of recombinant strains on the inflammatory response in macrophages. Relative mRNA expression levels of (**A**) *TNF-α*, (**B**) *IL-6*, and (**C**) *IL-1β* were assessed in RAW264.7 macrophages across different treatment groups. Gene expression was normalized to GAPDH and expressed relative to the Control group, which was set to 1.0. Data are presented as mean ± SEM (*n* = 6) and were analyzed by one-way ANOVA followed by Duncan’s multiple range test after confirmation of normality and homogeneity of variance. Different letters indicate significant differences (*p* < 0.05) between groups.

To evaluate the anti-inflammatory potential of *Lc. paracasei* L9 recombinants expressing Amuc_1100 protein, RAW264.7 macrophages were stimulated with LPS to induce pro-inflammatory responses. The effects of three recombinant strains—intracellular (pA-L9), secretory (pUA-L9), and surface-display (pUPA-L9)—on mRNA levels of *TNF-α*, *IL-6*, and *IL-1β* were analyzed (Figure 2A–C).

LPS treatment significantly upregulated *TNF-α*, *IL-6*, and *IL-1β* expression compared to the Control group (Figure 2A–C). All recombinant strains reduced LPS-induced cytokine expression relative to the LPS group. Notably, strains expressing Amuc_1100 protein exhibited enhanced anti-inflammatory activity compared to the *Lc. paracasei* L9 with empty vector (L9).

The recombinant strain expressing intracellular Amuc_1100 protein (pA-L9) significantly suppressed *TNF-α* and *IL-1β* levels compared with L9, but showed no significant effect on *IL-6* expression. In contrast, the recombinant *Lc. paracasei* L9 secreting Amuc_1100 protein (pUA-L9) markedly inhibited all three cytokines, with stronger *TNF-α* suppression than pA-L9. The recombinant strain displaying Amuc_1100 protein on the surface (pUPA-L9) significantly reduced *TNF-α* levels relative to pA-L9, but exhibited no statistical differences in *IL-6* and *IL-1β* regulation. Compared with pUA-L9, pUPA-L9 did not show additional inhibitory advantages for any of the tested cytokines. Notably, *A. muciniphila* (AKK) most potently suppressed *TNF-α*, outperforming all recombinant strains. However, its inhibitory effects on *IL-6* and *IL-1β* were not statistically significant when compared with pA-L9, pUA-L9, and pUPA-L9.

These findings underscore the varied anti-inflammatory effects of different Amuc_1100 expression systems, with pUA-L9 and pUPA-L9 showing the broadest cytokine inhibition among the engineered *Lc. paracasei*. Consequently, pUA-L9 and pUPA-L9 were selected to investigate their ameliorative effects on dextran sulfate sodium (DSS)-induced intestinal barrier damage and colitis in murine models.

### 3.3. Ameliorative Effects of Lc. paracasei L9 Recombinant Strains on DSS-Induced Colitis in Mice

**Figure 3:**
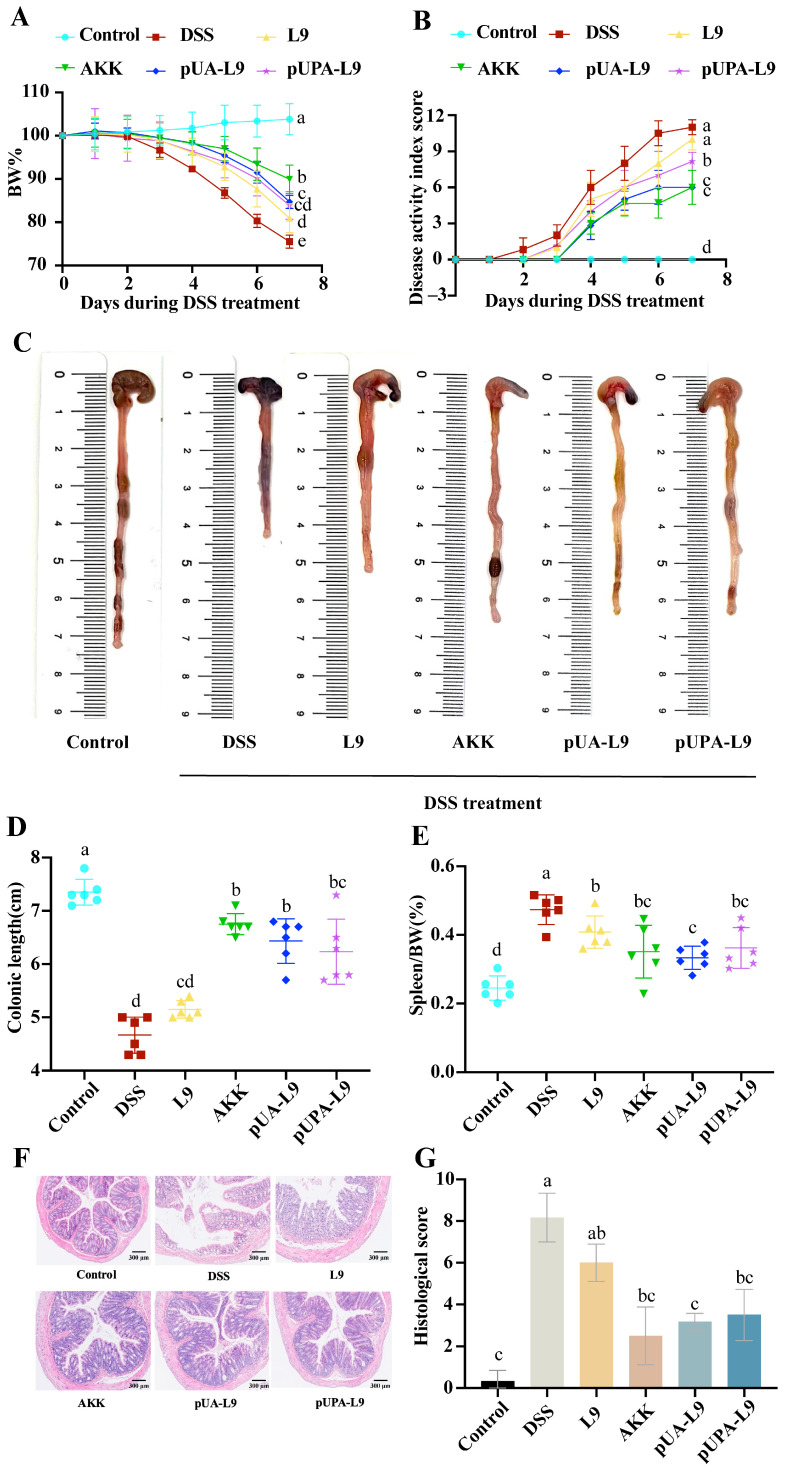
Effect of recombinant strains on disease symptoms and tissue injury of colitis mice. (**A**) Percentage change in body weight of mice during DSS modeling; (**B**) DAI scores of mice during modeling; (**C**) Representative images of mouse colons (unit: centimeter); (**D**) Mouse colon length (unit: centimeter); (**E**) Spleen as a percentage of body weight; (**F**) H&E staining of mouse colon sections; (**G**) Mouse colon tissue injury score. Data are presented as mean ± SEM (*n* = 6) and were analyzed by one-way ANOVA followed by Duncan’s multiple range test after confirmation of normality and homogeneity of variance. Different letters indicate significant differences (*p* < 0.05) between groups.

To evaluate the therapeutic potential of recombinant *Lc. paracasei* L9 expressing Amuc_1100 protein, a dextran sulfate sodium (DSS)-induced colitis murine model was established. Key parameters, including body weight, disease activity index (DAI), colon length, spleen weight, and histopathological changes, were analyzed (Figure 3A–G).

For weight loss and disease severity, DSS treatment elicited a notable 24.47 ± 1.40% reduction in body weight by day 7 alongside a corresponding elevation in DAI scores to 11.0 ± 0.63 within the DSS group—findings collectively indicative of acute colitis pathogenesis, as illustrated in Figure 3A,B. Almost all recombinant strains demonstrated notable attenuation of DSS-induced weight loss and disease severity when compared to the DSS group. Most recombinant strains expressing Amuc_1100 conferred a more pronounced therapeutic benefit than the empty vector control (L9). Moreover, the pUA-L9 exerted a stronger suppressive effect on disease activity index (DAI) scores than pUPA-L9. Notably, native *A. muciniphila* (AKK) administration markedly attenuated most disease parameters, including weight loss, disease activity index (DAI), and colon shortening.

For colon length and spleen weight, the administration of DSS induced remarkable shortening of the colon length (DSS group: 4.67 ± 0.34 cm vs. Control group: 7.35 ± 0.24 cm) and splenic edema. DSS-induced colon shortening and spleen enlargement (0.47 ± 0.04% of body weight) were markedly attenuated by pUA-L9 (Figure 3C–E). *Lc. paracasei* L9 displaying Amuc_1100 protein on the surface (pUPA-L9) showed no significant improvement in colon length or spleen weight compared to L9. H&E staining revealed severe colonic damage in DSS-treated mice, including crypt loss, epithelial disruption, and submucosal inflammatory infiltration (Figure 3F,G). Tissue injury scores were highest in the DSS group (8.17 ± 1.17), while the pUA-L9, pUPA-L9, and AKK significantly reduced damage. pUPA-L9 and AKK had no significant effect compared with L9. Remarkably, *Lc. paracasei* L9 secreting Amuc_1100 (pUA-L9) showed significantly lower histological scores compared to the empty vector control (L9).

The results showed that recombinant strains expressing Amuc_1100, particularly recombinant *Lc. paracasei* L9 secreting Amuc_1100 protein (pUA-L9), alleviated DSS-induced colitis by reducing weight loss, improving DAI scores, preserving colon length, mitigating spleen enlargement, and attenuating histopathological damage. These findings preliminarily suggested that the secretory delivery of Amuc_1100 (pUA-L9) might offer broad systemic benefits, while the surface-displayed form (pUPA-L9) showed a distinct profile, prompting further investigation into their mechanistic differences.

### 3.4. Modulation of Inflammatory Cytokines and NF-κB Signaling by Lc. paracasei L9 Recombinant Strains in Colitis Mice

**Figure 4:**
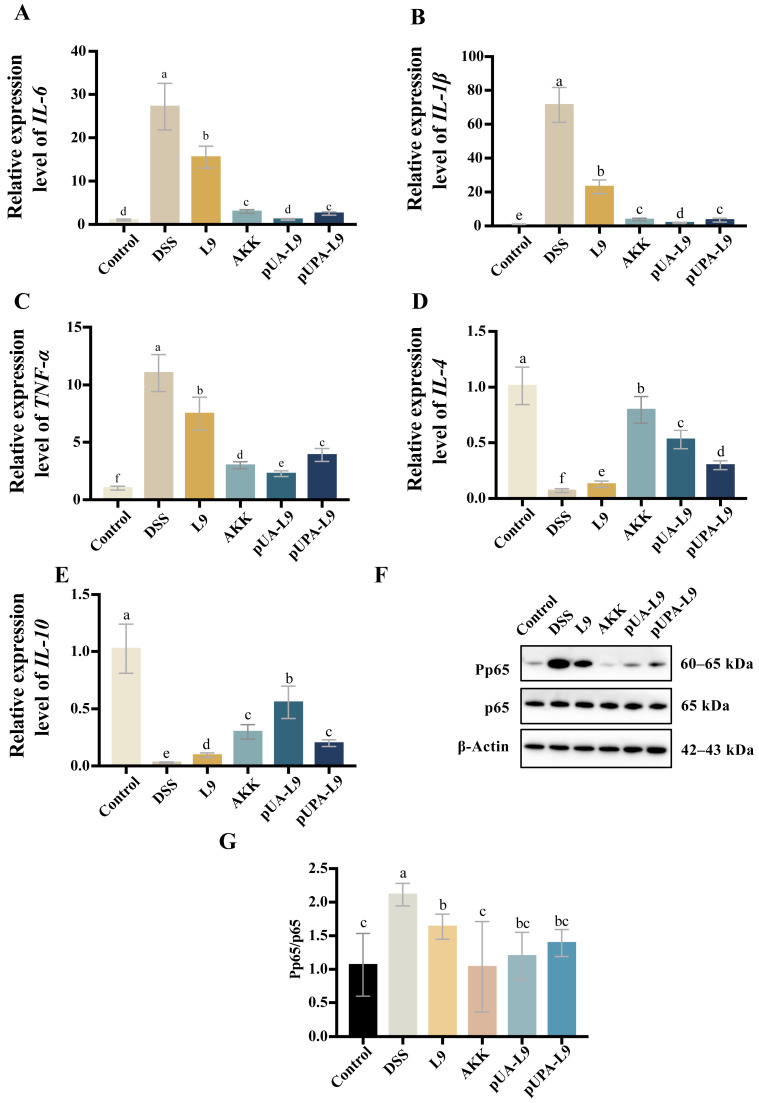
Effect of recombinant strains on the inflammatory response of colitis mice. Effect of different groups on the relative mRNA expression of (**A**) *IL-6*, (**B**) *TNF-α*, (**C**) *IL-1β*, (**D**) *IL-4* and (**E**) *IL-10* in mouse colon tissue. Gene expression was normalized to GAPDH and expressed relative to the Control group, which was set to 1.0. (**F**,**G**) Western blot and densitometry analysis of p65 and Pp65. Data are presented as mean ± SEM (*n* = 6) and were analyzed by one-way ANOVA followed by Duncan’s multiple range test after confirmation of normality and homogeneity of variance. Different letters indicate significant differences (*p* < 0.05) between groups.

To assess the anti-inflammatory mechanisms of recombinant *Lc. paracasei* L9 expressing Amuc_1100 protein, mRNA levels of pro-inflammatory cytokines (*IL-6*, *TNF-α*, and *IL-1β*) and anti-inflammatory cytokines (*IL-4* and *IL-10*) were measured in colon tissues of DSS-induced colitis mice (Figure 4A–E). Additionally, phosphorylated NF-κB p65 levels were analyzed via Western blot (Figure 4F,G).

DSS treatment significantly elevated pro-inflammatory cytokines (*IL-6*, *TNF-α*, and *IL-1β*) and suppressed anti-inflammatory cytokines (*IL-4* and *IL-10*) compared to the Control group (Figure 4A–E). All recombinant *Lc. paracasei* L9 and *A. muciniphila* (AKK) attenuated these effects. Compared to the *Lc. paracasei* L9 with empty vector (L9), recombinant *Lc. paracasei* L9 expressing Amuc_1100 protein (pUA-L9 and pUPA-L9) and AKK further reduced pro-inflammatory cytokines and enhanced *IL-4* and *IL-10* levels. Remarkably, Recombinant *Lc. paracasei* L9 secreting Amuc_1100 protein (pUA-L9) exhibited the strongest suppression of *IL-6*, *IL-1β*, and *TNF-α* (*p* < 0.05) and the highest induction of *IL-10* (Figure 4A–E). While recombinant *Lc. paracasei* L9 displaying Amuc_1100 protein on the surface (pUPA-L9) showed intermediate efficacy, with no significant advantage over pUA-L9.

DSS significantly increased phosphorylated NF-κB p65 levels, indicative of pathway activation. Recombinant strains and AKK markedly reduced phosphorylation compared to the DSS group (*p* < 0.05), with AKK showing the strongest inhibition (Figure 4F,G).

Collectively, these results established pUA-L9 as a potent systemic anti-inflammatory agent, primarily through the NF-κB pathway.

### 3.5. Restoration of Intestinal Tight Junction Proteins by Lc. paracasei L9 Recombinant Strains

**Figure 5:**
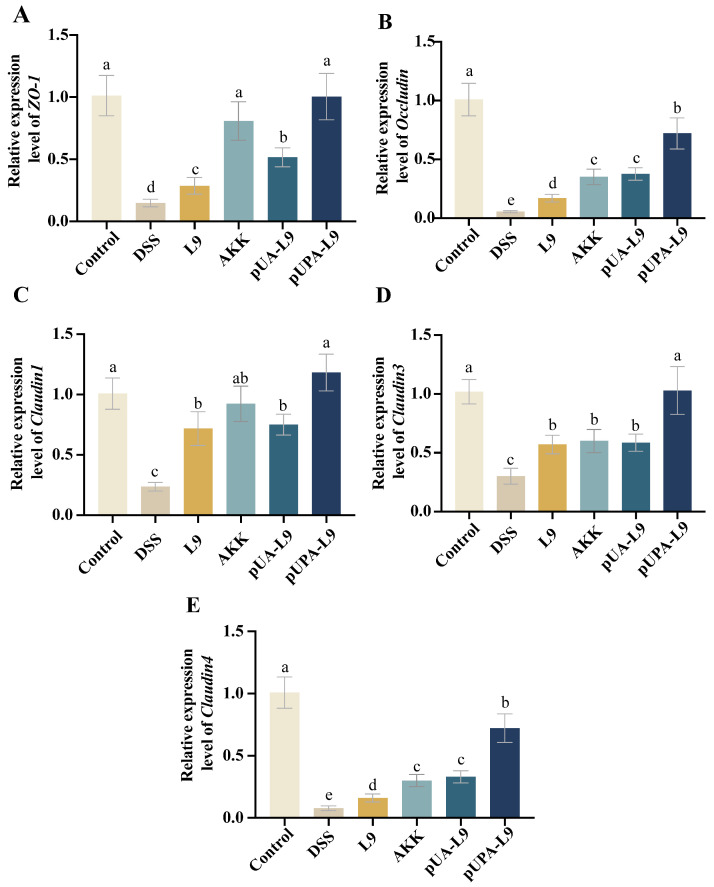
Effect of recombinant strains on the tight junction of colitis mice. Effect of different groups on the relative mRNA expression of (**A**) *ZO-1*, (**B**) *Occludin*, (**C**) *Claudin1*, (**D**) *Claudin3* and (**E**) *Claudin4* in mouse colon tissue. Gene expression was normalized to GAPDH and expressed relative to the Control group, which was set to 1.0. Data are presented as mean ± SEM (*n* = 6) and were analyzed by one-way ANOVA followed by Duncan’s multiple range test after confirmation of normality and homogeneity of variance. Different letters indicate significant differences (*p* < 0.05) between groups.

To evaluate the impact of recombinant *Lc. paracasei* L9 on intestinal barrier integrity, mRNA levels of tight junction proteins (*ZO-1*, *Occludin*, *Claudin1*, *Claudin3*, and *Claudin4*) were quantified in colon tissues (Figure 5A–E).

DSS treatment significantly downregulated all tight junction-associated markers compared to the Control group (*p* < 0.05). Recombinant *Lc. paracasei* L9 and *A. muciniphila* (AKK) significantly restored the expression levels of these tight junction-associated markers, as demonstrated in Figure 5A–E. Recombinant *Lc. paracasei* L9 secreting Amuc_1100 protein (pUA-L9) exhibited effects comparable to AKK, both significantly inhibited the reduction in *ZO-1*, *Occludin*, and *Claudin4* when compared to L9. Notably, recombinant *Lc. paracasei* L9, displaying Amuc_1100 protein on the surface (pUPA-L9) exhibited a pronounced advantage in upregulating expression of tight junction-associated markers, outperforming all recombinant strains and AKK.

As shown in the results, pUPA-L9 exhibited the strongest capacity to restore intestinal tight junctions, highlighting its role in preserving barrier function during colitis. This stark contrast underscores the concept that surface-display optimally positions Amuc_1100 to directly engage with and stabilize epithelial tight junctions.

### 3.6. Enhancement of Colonic Mucus Barrier by Lc. paracasei L9 Recombinant Strains

**Figure 6:**
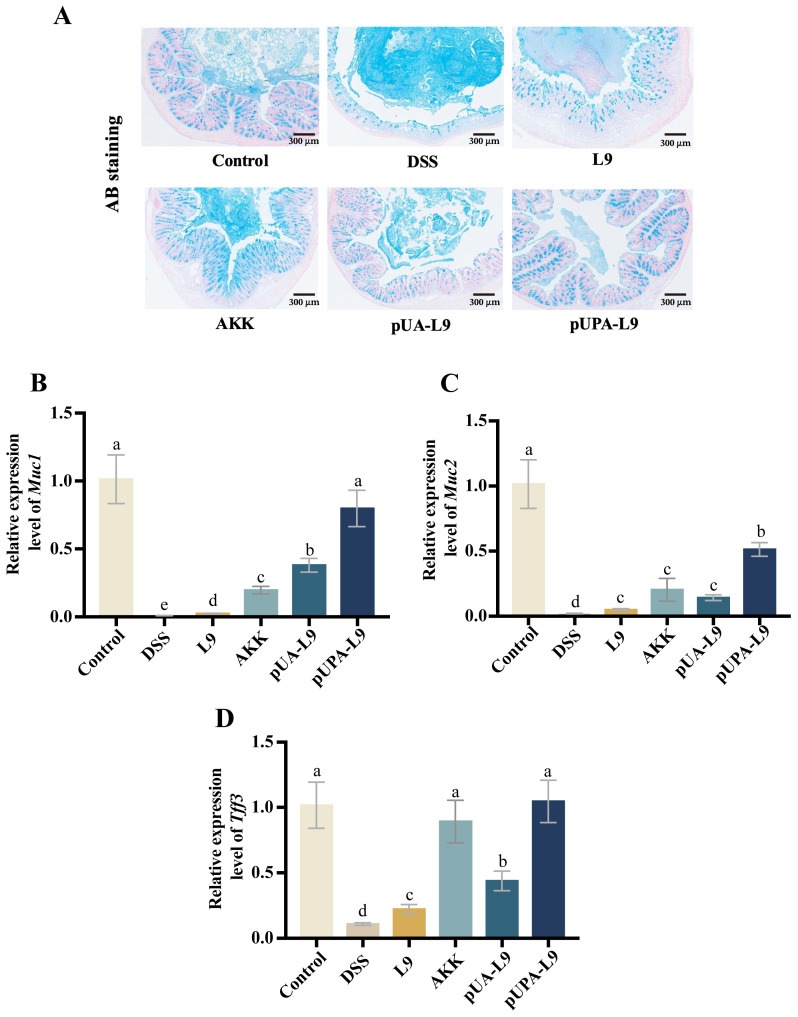
Effect of recombinant strains on the mucus barrier of colitis mice. (**A**) AB staining of mouse colon tissue sections. Effect of different groups on the relative mRNA expression of (**B**) *Muc1*, (**C**) *Muc2*, and (**D**) *Tff3* in mouse colon tissue. Gene expression was normalized to GAPDH and expressed relative to the Control group, which was set to 1.0. Data are presented as mean ± SEM (*n* = 6) and were analyzed by one-way ANOVA followed by Duncan’s multiple range test after confirmation of normality and homogeneity of variance. Different letters indicate significant differences (*p* < 0.05) between groups.

To evaluate the impact of recombinant *Lc. paracasei* L9 on the mucus barrier in DSS-induced colitis mice, Alcian Blue (AB) staining and gene expression quantification of mucin-associated markers (*Muc1*, *Muc2*, and *Tff3*) were performed (Figure 6A–D).

AB staining revealed a uniform, continuous mucus layer covering the colonic epithelium in the Control group. DSS treatment disrupted the mucus structure, resulting in a thickened, irregular, and unevenly stained layer. Recombinant *Lc. paracasei* L9 secreting Amuc_1100 protein (pUA-L9), *Lc. paracasei* L9 displaying Amuc_1100 protein on the surface (pUPA-L9) and *A. muciniphila* (AKK) restored mucus morphology and thickness, with pUPA-L9 showing the most pronounced improvement (Figure 6A).

DSS significantly suppressed *Muc1*, *Muc2*, and *Tff3* expression (*p* < 0.05) when compared with the Control group. All recombinant strains and AKK mitigated these reductions. Recombinant *Lc. paracasei* L9 displaying Amuc_1100 protein on the surface (pUPA-L9) exhibited the strongest efficacy, particularly in regulating *Muc1* and *Muc2* levels, outperforming other treatment groups. Compared to the *Lc. paracasei* L9 with empty vector (L9), recombinant strain secreting Amuc_1100 protein (pUA-L9) also demonstrated significant upregulation of both *Muc1* and *Tff3* expression, while AKK exhibited superior efficacy in *Muc2* expression to L9 and pUA-L9 (Figure 6B–D).

Together with the tight junction data, these findings demonstrate that pUPA-L9 improves local intestinal barrier function by enhancing both the chemical barrier (mucus-associated factors) and the physical barrier (tight junction–related markers) of the gut epithelium.

### 3.7. Regulation of Goblet Cell Differentiation and Maturation by Lc. paracasei L9 Recombinant Strains

**Figure 7:**
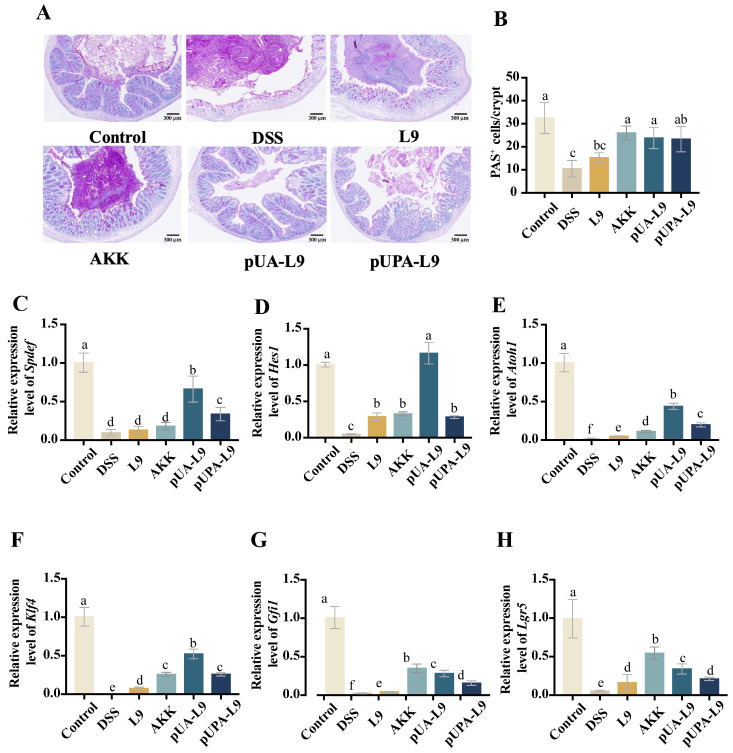
Effect of recombinant strains on differentiation and maturation of goblet cells. (**A**) PAS staining of mouse colon tissue sections. (**B**) Number of PAS^+^ goblet cells in each crypt. Effect of different groups on the relative mRNA expression of (**C**) *Spdef*, (**D**) *Hes1*, (**E**) *Atoh1*, (**F**) *Klf4*, (**G**) *Gfi1*, and (**H**) *Lgr5* in mouse colon tissue. Gene expression was normalized to GAPDH and expressed relative to the Control group, which was set to 1.0. Data are presented as mean ± SEM (*n* = 6) and were analyzed by one-way ANOVA followed by Duncan’s multiple range test after confirmation of normality and homogeneity of variance. Different letters indicate significant differences (*p* < 0.05) between groups.

The effects of recombinant *Lc. paracasei* L9 on goblet cell dynamics were assessed via Periodic Acid-Schiff (PAS) staining and gene expression analysis of differentiation-associated markers (*Spdef*, *Hes1*, *Atoh1*, *Klf4*, *Gfi1*, and *Lgr5*) (Figure 7A–H).

PAS staining revealed that DSS treatment led to a significant reduction in the number of PAS^+^ goblet cells, as well as induced morphological abnormalities of mouse colon tissue (Figure 7A,B). Recombinant *Lc. paracasei* L9 and *A. muciniphila* (AKK) partially restored cell numbers and morphology. Recombinant *Lc. paracasei* L9 secreting Amuc_1100 protein (pUA-L9) and AKK significantly increased PAS^+^ cells compared to *Lc. paracasei* L9 with empty vector (L9) (*p* < 0.05), while *Lc. paracasei* L9 displaying Amuc_1100 protein on the surface (pUPA-L9) showed modest, non-significant improvement (Figure 7B). Mechanistically, DSS downregulated Notch pathway regulators (*Spdef*, *Hes1*, *Atoh1*, *Klf4*, and *Gfi1*) and stem cell marker *Lgr5* (Figure 7C–H). Recombinant strains counteracted these effects. The pUPA-L9 significantly upregulated *Spdef*, *Atoh1*, *Klf4*, and *Gfi1* compared to L9 (*p* < 0.05), surpassing AKK in *Spdef*, *Atoh1*, and *Klf4* regulation. pUA-L9 exhibited the strongest efficacy, particularly in regulating *Spdef*, *Hes1*, *Atoh1*, and *Klf4* levels, outperforming other treatment groups. Notably, the induction of *Hes1* expression by pUA-L9 was approximately fourfold higher than that observed in the pUPA-L9 group, while both pUA-L9 and pUPA-L9 restored *Lgr5* expression, with pUA-L9 showing stronger efficacy than pUPA-L9 (Figure 7F).

This unique ability of pUA-L9 to promote goblet cell differentiation and epithelial regeneration, likely mediated by soluble signaling, complements its systemic anti-inflammatory effects.

## 4. Discussion

This study investigates whether the subcellular localization of the postbiotic protein Amuc_1100 within a probiotic chassis influences its therapeutic profile in DSS-induced colitis. By expressing Amuc_1100 intracellularly (pA-L9), extracellularly (pUA-L9), or as a surface-displayed protein (pUPA-L9) in *Lc. paracasei* L9, we aimed to explore whether different delivery strategies are associated with distinct biological outcomes *in vivo*. Rather than demonstrating categorical superiority of one strategy over another, our findings suggest that secretory and surface-display expression of Amuc_1100 confer largely comparable overall efficacy, while exhibiting different functional tendencies across immune modulation and epithelial barrier–related endpoints.

Consistent with previous reports highlighting the advantages of most lactic acid bacteria (LAB) as delivery vehicles for therapeutic proteins, *Lc. paracasei* L9 represents an appropriate and well-established probiotic chassis for intestinal delivery applications [46,47,48,49,50,51,52]. LAB are known to exhibit intrinsic resistance to gastrointestinal stressors and well-documented immunomodulatory properties, making them suitable hosts for proof-of-concept studies investigating the spatial delivery of bioactive molecules. While *Lactococcus lactis* is frequently used as a recombinant chassis, its limited colonization capacity contrasts with the broader metabolic and ecological adaptability of *Lactobacillus* species, including *Lc. paracasei* L9 [53,54,55].

Three primary strategies exist for recombinant protein expression in lactic acid bacteria (LAB): intracellular retention, extracellular secretion, and surface display. These approaches differ fundamentally in protein localization, yield, bioactivity, and *in vivo* efficacy. Regarding protein yield, we further confirmed that the three expression strategies differed significantly. The recombinant strain expressing Amuc_1100 intracellularly (pA-L9) produced the highest protein levels, followed by the secretory expression system (pUA-L9), whereas the surface-displayed expression system (pUPA-L9) yielded the lowest amount of Amuc_1100 (Figure S2). Previous studies have demonstrated that different heterologous expression patterns affect the biological functions of proteins. For instance, Bahey-El-Din et al. demonstrated that secretory expression of *Listeria monocytogenes* lysin O (LLO) in *Lactococcus lactis* elicited stronger antigen-specific immune responses compared to intracellular expression [56]. Similarly, Bermúdez-Humarán et al. reported that secretory delivery of human papillomavirus E7 protein in *Lactococcus lactis* enhanced mucosal immunity more effectively than surface display [57]. Building on these precedents, our study revealed that both secretory (pUA-L9) and surface-display (pUPA-L9) strategies significantly suppressed lipopolysaccharide (LPS)-induced pro-inflammatory cytokines (*TNF-α*, *IL-6*, and *IL-1β*) in macrophages. Both strategies outperformed intracellular expression in this regard. This divergence may stem from the limited capacity of intracellular proteins to directly engage host cells via surface interactions [56].

In the DSS-induced colitis model, both secretory and surface-displayed strains alleviated weight loss, disease activity index elevation, colon shortening, splenomegaly, and histological injury, with no consistent statistically significant differences between pUA-L9 and pUPA-L9 across global disease indices. Nevertheless, domain-specific trends emerged. pUA-L9 tended to associate more strongly with reduced inflammatory cytokine expression and transcriptional markers linked to goblet cell differentiation (e.g., *Spdef*, *Hes1*, *Atoh1*, and *Klf4*), whereas pUPA-L9 showed relatively higher expression of tight junction– and mucus-associated genes *(Muc1*, *Muc2*, *Occludin*, *Claudin3*, and *Claudin4*).

The preferential upregulation of barrier-associated markers observed with pUPA-L9 is biologically plausible in light of the known adhesive properties of Amuc_1100. This protein is a highly abundant fimbrial-like outer membrane component of *A. muciniphila* and has been closely linked to bacterial adhesion [22,58]. Previous studies have shown that surface-displayed adhesins, including Amuc_1100, can bind O-glycans within mucins, facilitating the formation of biofilm-like structures that may enhance bacterial retention within the mucus layer and reduce clearance by intestinal peristalsis [59]. Consistent with this concept, surface modification strategies—such as polyethylene glycol (PEG) coating—have been reported to increase mucosal retention of commensal bacteria *in vivo* [59]. Similarly, incorporation of selenium dots into the bacterial envelope of *Lc. casei* enhanced therapeutic efficacy in ulcerative colitis models, underscoring the potential benefit of surface-associated modifications in strengthening host–microbe interactions [60]. Zhang et al. further demonstrated that surface-displayed Amuc_1100 contributes to improved tight junction integrity, supporting a role for adhesion-mediated local effects on the epithelial barrier [61].

In contrast, the secretory strain pUA-L9 was more consistently associated with systemic anti-inflammatory readouts, including reduced cytokine expression and transcriptional signatures related to epithelial renewal and goblet cell differentiation. A plausible explanation is that secreted Amuc_1100, as a soluble factor, may diffuse beyond the immediate epithelial surface and interact with immune cells or crypt-resident populations. Supporting this interpretation, Amuc_1100 has been shown to activate TLR2 and TLR4 signaling pathways in peripheral blood mononuclear cells, leading to modulation of cytokine production, including *IL-10*, *IL-6*, *IL-8*, and *TNF-α* [25]. Nevertheless, these mechanistic considerations remain inferential, as direct evidence for differential spatial distribution, cellular targeting, or retention of secreted versus surface-anchored Amuc_1100 was not obtained in the present study.

Taken together, these findings suggest that subcellular localization of Amuc_1100 within a probiotic vector may bias therapeutic outcomes toward either systemic immunomodulation or localized barrier-associated responses, without conferring absolute or categorical superiority of one strategy over the other. Rather than defining rigid functional specialization, our data support a model in which secretory and surface-display delivery modes generate partially overlapping but functionally skewed response profiles. This conceptual framework provides a basis for tailoring probiotic delivery strategies according to disease stage—for example, favoring secretory constructs during acute inflammatory phases and surface-displayed constructs during barrier repair and remission. More broadly, the concept of spatial functionality may be applicable to other therapeutic proteins, offering a route toward more precisely engineered probiotic interventions.

Although pUPA-L9 markedly increased the expression of tight junction- and mucus-associated markers, direct functional measurements of epithelial barrier integrity (such as TEER or FITC-dextran permeability) were not performed in this study and warrant further investigation. Moreover, while our data suggest that the therapeutic effects of Amuc_1100 are spatially regulated by its subcellular localization, direct evidence of protein distribution—such as fluorescent tagging—will be required to conclusively validate the proposed spatial mechanisms of action. Further validation using human gut organoids or clinical studies will be essential to assess the translational potential and scalability of this delivery strategy. In addition, future studies should explore combinatorial expression approaches, such as co-expression of secretory and surface-displayed Amuc_1100, to achieve synergistic anti-inflammatory and barrier-restorative effects.

Despite the promising therapeutic outcomes observed in this study, important biosafety and translational considerations remain before clinical or food-related applications can be realized. In the present work, antibiotic-resistance–bearing plasmids were employed to ensure stable expression of Amuc_1100 and to enable systematic comparison of subcellular localization strategies. While appropriate for proof-of-concept studies, the use of antibiotic selection markers raises concerns regarding horizontal gene transfer within the gut microbiota and presents regulatory barriers for live biotherapeutic products and functional foods [46,47]. Importantly, these limitations arise from the current genetic implementation rather than the spatial delivery concept itself. In future development, antibiotic markers could be replaced with food-grade or marker-free systems, and chromosomal integration of the Amuc_1100 expression cassette could enhance genetic stability and minimize horizontal gene transfer. Moreover, combining chromosomal integration with inducible or environmentally responsive promoters may further improve safety and regulatory compliance, thereby facilitating the translational application of spatially targeted probiotic therapies.

## 5. Conclusions

This study shows that heterologous expression of Amuc_1100 in *Lacticaseibacillus paracasei* L9 improves intestinal barrier function and mitigates DSS-induced colitis. Our data indicate that distinct expression strategies are associated with different functional outcomes: secretory expression (pUA-L9) is primarily linked to systemic anti-inflammatory responses and enhanced goblet cell differentiation, whereas surface display (pUPA-L9) preferentially supports local barrier integrity by restoring mucus layer organization and tight junction expression. Together, these findings underscore the relevance of protein spatial localization within probiotic chassis systems and suggest that spatially targeted probiotic delivery represents a promising and modular strategy for the development of precision interventions for inflammatory bowel disease.

## Data Availability

The original contributions presented in the study are included in the article/Supplementary Material, further inquiries can be directed to the corresponding author.

## Supplementary Materials

The following supporting information can be downloaded at: https://www.mdpi.com/article/10.3390/nu18010123/s1, The information of bacterial strains and plasmids (Table S1, [62,63,64]); the primers used for PCR (Table S2); the scoring system of disease activity index (DAI) (Table S3); criteria for histological scores of colon damage (Table S4); the primers used for qRT-PCR from Raw264.7 (Table S5); the primers used for qRT-PCR from mice (Table S6); The schematic diagram of animal experiment design (Figure S1); Relative concentration of Amuc_1100 protein in different recombinant strains (Figure S2); H&E staining of mouse colon sections (Figure S3).

## Abbreviations

The following abbreviations are used in this manuscript:

AB	Alcian Blue
AhR	aryl hydrocarbon receptor
BW	body weight
CD	Crohn’s disease
CTLs	CD8+ cytotoxic T lymphocytes
DAI	disease activity index
DSS	dextran sulfate sodium
GBD	Global Burden of Disease
GRAS	Generally Recognized As Safe
IACUC	Institutional Animal Care and Use Committee
IBD	inflammatory bowel disease
LAB	Lactic acid bacteria
LPS	Lipopolysaccharide
SCFA	short chain fatty acid
SPF	specific pathogen free
TEM	transmission electron microscopy
TLR2	Toll-like receptors 2
TLR4	Toll-like receptors 4
PAS	Periodic Acid-Schiff
PBMCs	peripheral blood mononuclear cells
PEG	polyethylene glycol
PVDF	polyvinylidene fluoride
UC	ulcerative colitis
