# Supplementary material for: Subcellular Localization Dictates Therapeutic Function: Spatially Targeted Delivery of Amuc_1100 by Engineered Lacticaseibacillus paracasei L9 Enhances Intestinal Barrier in Colitis
Source: Nutrients. 2025 Dec 30;18(1):123. doi: 10.3390/nu18010123 (PMC12787787; doi:10.3390/nu18010123)
Supplement: Supplementary file 1 [file nutrients-18-00123-s001.zip › nutrients-4024624-supplementary.pdf]

Supporting information

Subcellular Localization Dictates Therapeutic Function: Spatially Targeted Delivery of Amuc_1100 by Engineered *Lacticaseibacillus paracasei* L9 Enhances Intestinal Barrier in Colitis

Table S1 Bacterial Strains and Plasmids

Strains and plasmids	Description	Source
Lacticaseibacillus paracasei L9	Host strain, isolated from the gut of healthy centenarian	[62]
Lactococcus lactis NZ9000	Intermediate host bacteria expressing Amuc_1100 protein	[63]
Akkermansia muciniphila ATCC BAA-835	Used as a control in experiment	American Type Culture Collection
pSIP411	spp-based inducible expression vector; SH71rep; EryR; PorfX: gusA	[64]

Table S2 Sequences of primer used for PCR

Gene	Primer
Am-F-411	5′-GGAGATTTTAGCCATGGGAATTGTTAATAGTAAACGCAG-3′
usp45-F-411	5′-ACAAGGAGATTTTAGCCATGGGAAAAAAAAAGATTATCTCAG-3′
usp45-R-Am	5′-CACTGCGTTTACTATTAACAATAGCGTAAACACCTGAC-3′
Am-F-usp45	5′-GTCAGGTGTTTACGCTATTGTTAATAGTAAACGCAGTG-3′
Am-R-411	5′-CACGTGCTGTAATTTGAAGCTTTTAATGATGATGATGATGATGATCC-3′
usp45-R-pgsA′	5′-GAAAGCTCAGTTCTTTTTCATAGCGTAAACACCTGACAACG-3′
pgsA′-F-usp45	5′-CGTTGTCAGGTGTTTACGCTATGAAAAAGAACTGAGCTT-3′
pgsA′-R-Am	5′-CACTGCGTTTACTATTAACAATGAGAACCGTGAAATTCATATCC-3′
Am-F-pgsA′	5′-GGATATGAATTTCACGGTTCTCATTGTTAATAGTAAACGCAGTG-3′
411-Seq-F	5′-CGCCTTAGTGCCTTAGTTTGTG-3′
411-Seq-R	5′-CCCGCCCTTATGGGATTT-3′

Table S3 Disease activity index (DAI) scoring system

Score	Body weight loss (BW %) [a]	Stool Consistency	Occult blood in stool[b]
0	None	Hard, pellet - like stools	None
1	1-5 BW	Soft stools	Occult blood in stool positive (+)
2	6-10 BW	Unformed soft or loose stools	Occult blood in stool positive (++)
3	11-18 BW	Watery stools	Occult blood in stool positive (+++)
4	>18 BW		Visible rectal bleeding

[a]The body weight (BW) loss of each mouse was expressed as a percentage relative to its baseline weight before DSS

treatment.

[b]The hemoccult positive degree was assessed by using the Fecal Occult Blood Test Kit (Solarbio, Beijing, China) based on color change.

Table S4 Histological scores of colon damage

Score	Inflammation severity	Inflammation extent	Crypt damage
0	None	None	Intact crypt, no damage
1	Mild inflammation with slight lesion sites	Inflammation observed only in mucosa	Basal 1/3 crypt damage
2	Moderate inflammation with several lesion sites	Inflammation observed in mucosa and submucosa positions	Basal 2/3 crypt damage
3	Severe inflammation with whole colonic damage	Inflammation observed in transmural colonic tissues	Crypt lost but surface epithelium present
4			Crypt and surface epithelium lost

Table S5 Sequences of primer used for Real-time PCR from Raw264.7

Gene	Primer (5′→3′)
GAPDH	Forward：AGGTCGGTGTGAACGGATTTG<br>Reverse：GGGGTCGTTGATGGCAACA
TNF-α	Forward：CTGAACTTCGGGGTGATCGG<br>Reverse：GGCTTGTCACTCGAATTTTGAGA
IL-1β	Forward：TTCAGGCAGGCAGTATCACTC<br>Reverse：GAAGGTCCACGGGAAAGACAC
IL-6	Forward：CTGCAAGAGACTTCCATCCAG<br>Reverse：AGTGGTATAGACAGGTCTGTTGG

Table S6 Sequences of primer used for Real-time PCR from mice

Gene	Primer (5′→3′)
GAPDH	Forward：AGGTCGGTGTGAACGGATTTG<br>Reverse：GGGGTCGTTGATGGCAACA
Muc1	Forward：GCTCCTTTCTTCCTGCTGCTA<br>Reverse：TGCTTCTGGCTCATTTATTCCT
Muc2	Forward：AGGGCTCGGAACTCCAGAAA<br>Reverse：CCAGGGAATCGGTAGACATCG
Tff3	Forward：CATCCATACTCCCTTGTCCAGA<br>Reverse：TGTGCTTTGCCTCCGTTCA
Spdef	Forward：ATGGACAGAACACCAGTACCG<br>Reverse：AGGCGCAGTAGTGAAGGGT
Hes1	Forward：TCAACACGACACCGGACAAAC<br>Reverse：ATGCCGGGAGCTATCTTTCTT
Atoh1	Forward：ACTGTCCCTCCTGGATAGCAC<br>Reverse：TGTTGAAGGACGGGATAACG
Klf4	Forward：GCCATCGGACCTACTTATCTGC

	Reverse：TTCCCTCCAATCCTCACCC
Gfi1	Forward：AGAAGGCGCACAGCTATCAC
	Reverse：GGCTCCATTTTCGACTCGC
Lgr5	Forward：GAGGAGGAGGAGGAGGAGGAG
	Reverse：CTGTGGAGTCCATCAAAGCA
ZO-1	Forward：GCCGCTAAGAGCACAGCAA
	Reverse：TCCCCACTCTGAAAATGAGGA
Occludin	Forward：TTGAAAGTCCACCTCCTTACAGA
	Reverse：CCGGATAAAAAGAGTACGCTGG
Claudin1	Forward：GGGGACAACATCGTGACCG
	Reverse：AGGAGTCGAAGACTTTGCACT
Claudin3	Forward：ACCAACTGCGTACAAGACGAG
	Reverse：CAGAGCCGCCAACAGGAAA
Claudin4	Forward：GTCCTGGGAATCTCCTTGGC
	Reverse：TCTGTGCCGTGACGATGTTG
IL-6	Forward：TAGTCCTTCCTACCCCAATTTCC
	Reverse：TTGGTCCTTAGCCACTCCT
IL-1β	Forward：TTCAGGCAGGCAGTATCACTC
	Reverse：GAAGGTCCACGGGAAAGACAC
TNF-α	Forward：GACGTGGAACTGGCAGAAGAG
	Reverse：TTGGTGGTTTGTGAGTGTGAG
IL-4	Forward：CCCCAGCTAGTTGTCATCCTG
	Reverse：CAAGTGATTTTTGTCGCATCCG
IL-10	Forward：CTTACTGACTGGCATGAGGATCA
	Reverse：GCAGCTCTAGGAGCATGTGG

Gene Sequence

Amuc_1100 protein（After codon optimization）:

ATGAGCAACTGGATTACCGATAATAAACCAGCCGCTATGGTTGCTGGCGTTGGCTTGTTGTTGTTT
TTGGGCTTGAGTGCCACCGGCTATATTGTTAATAGTAAACGCAGTGAACTGGATAAGAAGATTAG
CATTGCCGCTAAAGAAATCAAAAGTGCCAATGCCGCTGAAATCACCCCAAGTCGCAGTAGTAAT
GAAGAATTGGAAAAAGAATTGAACCGCTATGCCAAAGCCGTTGGCAGTTTGGAAACCGCCTATA
AACCATTTTTGGCCAGTAGTGCTTTGGTTCCAACCACGCCAACCGCCTTTCAAAATGAATTGAAA
ACCTTTCGCGATAGCTTGATTAGCAGCTGCAAGAAGAAGAACATTTTGATTACGGATACCAGTAG
CTGGTTGGGCTTTCAAGTTTATAGTACCCAAGCTCCAAGTGTTCAAGCCGCTAGTACCTTGGGCTT
TGAATTGAAGGCCATTAATAGCTTGGTTAACAAATTGGCCGAATGCGGCTTGAGCAAGTTTATTA
AGGTTTATCGCCCACAGTTGCCAATTGAAACCCCAGCCAATAATCCGGAAGAAAGTGATGAAGC
CGATCAAGCCCCATGGACGCCAATGCCATTGGAAATTGCCTTTCAAGGCGATCGTGAAAGTGTTT
TGAAAGCCATGAATGCCATTACCGGCATGCAGGATTATTTGTTTACCGTTAATAGCATTCGCATTC
GCAATGAACGCATGATGCCACCACCAATTGCCAATCCAGCCGCTGCCAAACCAGCTGCCGCTCA
ACCAGCCACCGGTGCCGCTAGTTTGACCCCAGCCGATGAAGCTGCTGCCCCAGCCGCTCCAGCC
ATTCAACAAGTTATTAAACCGTATATGGGCAAAGAACAGGTCTTTGTTCAAGTCAGCTTGAACTT
GGTCCATTTTAATCAACCAAAAGCTCAAGAACCGAGCGAGGATTAA

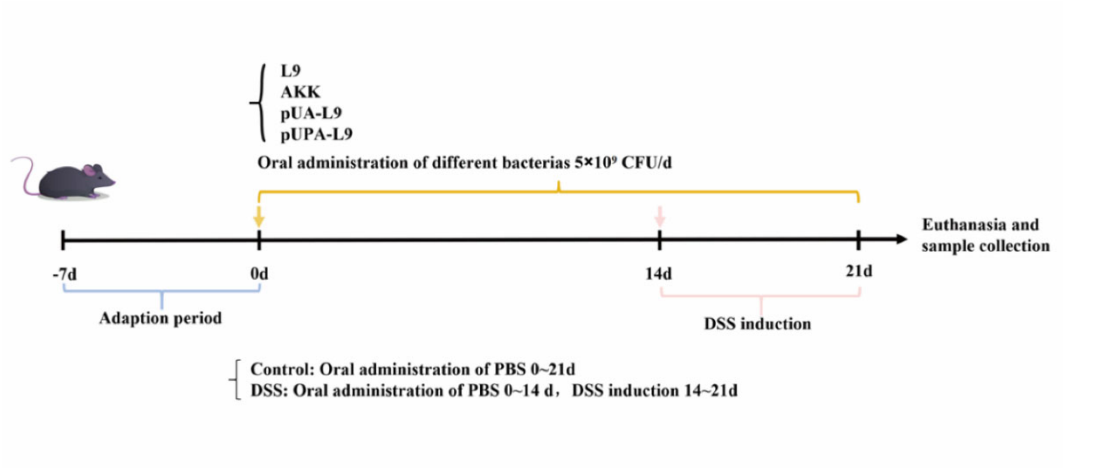

Figure S1 Schematic diagram of animal experiment design. mice were randomly allocated to six experimental groups (n = 6 biologically independent samples in each group) as follows: healthy mice consumed regular water (Control), model mice consumed 2.5% DSS (DSS), experimental mice consumed 2.5% DSS and administered *Lc. paracasei* L9 with empty vector (L9), experimental mice consumed 2.5% DSS and administered *A. muciniphila* (AKK), experimental mice consumed 2.5% DSS and administered *Lc. paracasei* L9 secreting Amuc_1100 protein (pUA-L9), experimental mice consumed 2.5% DSS and administered *Lc. paracasei* L9 displaying Amuc_1100 protein on the surface (pUPA-L9).

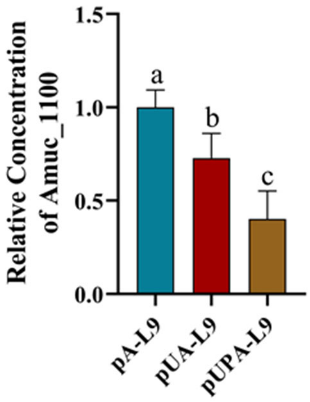

Figure S2 Relative concerntration of Amuc_1100 protein in different recombinant strains. Data are presented as the mean ± SEM (n = 3). Data were tested for normality using the Shapiro–Wilk test and for homogeneity of vari-ances prior to statistical analysis. When these assumptions were satisfied, differences among multiple groups were analyzed using one-way analysis of variance (ANOVA). Post-hoc comparisons were performed using Duncan’s multiple range test to identify dif-ferences between groups.

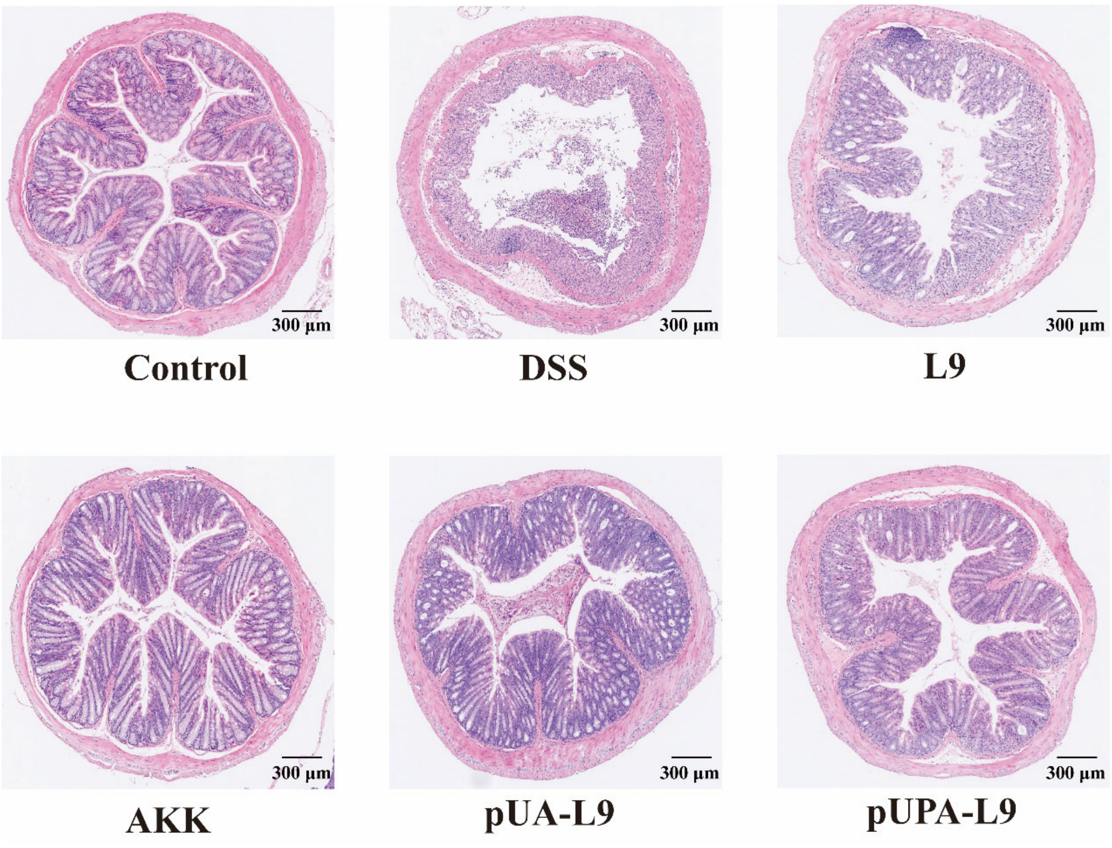

Figure S3 H&E staining of mouse colon sections.
